# Automating updates for scoping reviews on the environmental drivers of human and animal diseases: a comparative analysis of AI methods

**DOI:** 10.3389/frai.2025.1526820

**Published:** 2025-06-10

**Authors:** Rémy Decoupes, Claudia Cataldo, Luca Busani, Mathieu Roche, Maguelonne Teisseire

**Affiliations:** ^1^Territoires, environnement, télédétection et information spatiale (TETIS), Univ. Montpellier, AgroParisTech, Centre de coopération internationale en recherche agronomique pour le développement (CIRAD), CNRS, Institut national de recherche pour l'agriculture, l'alimentation et l'environnement (INRAE), Montpellier, France; ^2^INRAE, Montpellier, France; ^3^Center for Gender-Specific Medicine, Istituto Superiore di Sanitá, Rome, Italy; ^4^CIRAD, Montpellier, France

**Keywords:** scoping review, natural language processing (NPL), large language models (LLM), artificial intelligence (AI), infectious diseases, covariates analysis

## Abstract

Understanding the environmental factors that facilitate the occurrence and spread of infectious diseases in animals is crucial for risk prediction. As part of the H2020 Monitoring Outbreaks for Disease Surveillance in a Data Science Context (MOOD) project, scoping literature reviews have been conducted for various diseases. However, pathogens continuously mutate and generate variants with different sensitivities to these factors, necessitating regular updates to these reviews. In this paper, we propose to evaluate the potential benefits of artificial intelligence (AI) for updating such scoping reviews. We thus compare different combinations of AI methods for solving this task. These methods utilize generative large language models (LLMs) and lighter language models to automatically identify risk factors in scientific articles.

## 1 Introduction

The emergence of infectious diseases, especially those involving animal hosts and/or vectors, is significantly influenced by environmental factors that can directly impact the pathogens, their vectors, their reservoir species, their hosts, and humans alike (Giesen et al., 2023). Consequently, gaining a deeper understanding of these factors across the transmission chain becomes crucial (Cataldo et al., 2023) for understanding disease dynamics and the consequent risks faced by humans and animals. Structured literature searches and evidence syntheses are commonly used to obtain this knowledge and identify the significance (both positive and negative) of the observed factors (Sutton et al., 2019). The retrieved information is combined with relevant epidemiological data to inform predictive models (Wongnak et al., 2022). This is among the outcomes of the Monitoring Outbreaks for Disease Surveillance in a Data Science Context (MOOD) project (grant agreement no. 874850), which focuses on a list of prototype diseases and develops data collection and analysis systems and tools to support the early detection, assessment, and monitoring of the current and potential infectious disease threats in Europe and beyond.

Given the exponential surge in the production of global scientific literature and the relentless pace of global changes (such as climate and environmental condition changes and human, animal, and vector population changes), together with the emergence of new pathogens and variants, the rapid obsolescence of information is a pressing issue. Therefore, updating the information gained from a scoping review of the scientific literature in a timely manner is not just a necessity but also an urgent imperative in the face of these evolving circumstances.

However, scoping reviews and evidence syntheses of the scientific literature are time-consuming undertakings, hindering the pace at which the information is updated. Our research proposes a comparison among methods stemming from the latest advances in artificial intelligence (AI), particularly those focused on generative large language models (LLMs). These methods are not only promising but also offer opportunities for automating the update process of scoping reviews. By accelerating the pace at which new evidence can be synthesized and incorporated, they have the potential to revolutionize our understanding of the environmental factors that affect disease transmission.

To effectively train and evaluate these AI models, we utilize a dataset annotated by experts in human and animal health epidemiology, environmental science, and microbiology. These annotations, conducted at both the sentence and article levels, aim to extract the environmental conditions that impact the spread of epidemics. Our initial focus is on training the models related to influenza that use data, but we also assess their performance on datasets related to other infectious diseases, such as leptospirosis and chikungunya. This comprehensive evaluation conducted across different diseases demonstrates the potential applicability and scalability of automated approaches for updating systematic reviews in human and animal health.

## 2 Related work

The research direction to which our study contributes is the automation of structured literature reviews with a focus on scoping reviews. The main scoping review process can be divided into five main stages: searching for new references, screening (ranking articles by relevance), extracting data (extracting data or information relevant to the review question), assessing the quality of articles, and formatting the obtained results (Tsafnat et al., 2014; Marshall and Wallace, 2019). While various efforts have been made toward automating different aspects of scoping reviews, our proposal specifically targets the data extraction stage.

Automating the data extraction task presents a significant opportunity to streamline the scoping review process, as this stage often involves labor-intensive tasks such as extracting relevant information from a large volume of scientific literature. By leveraging advancements in AI and natural language processing (NLP), our study aims to develop and evaluate automated methods for extracting key data points from research articles that are relevant to the environmental factors influencing the transmission of infectious diseases in human and animals. Our focus on data extraction aligns with the recent trends in systematic review automation research: recognizing the potential of AI-driven approaches to improve the efficiency and accuracy of this critical stage.

While our proposal does not aim to fully automate the entire scoping review process, focusing specifically on data extraction allows us to address a key bottleneck and lay the foundation for future advancements toward comprehensive automation. By building upon existing methodologies and exploring novel AI techniques, our study contributes to the ongoing efforts to harness technology for enhancing the evidence synthesis and decision-making processes in the field of human and animal health.

Two types of tools are available for addressing this data extraction issue. The first type, semiautomatic tools, highlight sentences potentially containing interesting data; an example is the ExaCT tool (Kiritchenko et al., 2010), which is a sentence classifier. The second strategy involves performing automatic extraction through an NLP task known as named-entity recognition (NER) (Marshall et al., 2016). However, these tools have been trained for randomized controlled trials (RCTs) (Marshall and Wallace, 2019). Unfortunately, the question addressed in our review lies beyond this clinical and medical scope, making them unsuitable for reuse in our study, which aims to detect environmental factors.

This research direction that was highly popular in the mid-2010s appears to have received less attention since then. The prolific research conducted during that time led to the creation of a collection of tools cataloged on the SR Toolbox website (Marshall and Brereton, 2015). Unfortunately, these software solutions, which were developed by groups of researchers, face challenges in terms of maintenance. The reuse of these approaches a few years later is not always feasible. Moreover, the field of NLP has evolved rapidly since 2017 with the advent of attention-based models and transformer architectures (Vaswani et al., 2017) and since November 2022 with the launch of ChatGPT, which has been widely used by non-NLP experts for a variety of tasks.

Indeed, since 2019, numerous pretrained language models, such as bidirectional encoder representations from transformers (BERT) (Devlin et al., 2019), RoBERTa (Liu et al., 2019), and XLM-RoBERTa (Conneau et al., 2020), have been made available to the community. These models are also referred to as small language models (SLMs) due to their sizes relative to those of newer models such as ChatGPT, or they can be alternatively referred to as masked language models (MLMs) because of the name of their pretraining task; such models can be easily fine-tuned for addressing a specific task and domain. An example is the Microsoft BiomedBERT (Gu et al., 2022) model adapted for the biomedical domain. These pre-trained models, whether general or specific, can be fine-tuned to detect mentions of environmental risk factors through an NER task.

However, to truly improve these models for solving a particular task, one must be able to compile a substantial training dataset, i.e., at least 10,000 data points (Bayer et al., 2022). This requires launching an annotation campaign involving epidemiology experts in animal healthcare. Gathering such an amount of data is inconceivable. Therefore, various techniques can be used to artificially increase the size of the utilized training dataset, either through data augmentation (Wei and Zou, 2019; Longpre et al., 2020; Feng et al., 2021; Nie et al., 2020; Dai et al., 2023) or semisupervised methods (Chen et al., 2020; Shams, 2014). The difference between these two types of approaches is that the former creates new annotated sentences, whereas the latter involves automatically labeling sentences that are not manually annotated.

Finally, since 2022, the NLP field has witnessed the emergence of LLMs. What sets them apart from MLMs is their numbers of parameters (exceeding 1 billion), as well as the sizes of their pretraining datasets (exceeding one trillion tokens). These models specialize in text generation tasks; however, with a suitable prompt, it is possible to make them perform an NER task.

Similar to MLMs, LLMs need guidance to detect new types of labels, such as those examined in our study. For this purpose, different techniques are available. The first, few-shot learning (Brown et al., 2020), involves incorporating a series of examples into the constructed prompt (sentences with tokens labeled as risk factors, for example). The second, retrieval-augmented generation (RAG) (Lewis et al., 2021), involves creating a knowledge base for the domain in the form of vectorized data embeddings of relevant phrases. An embedding is the vector representation of a word or group of words formed by a model such as word2vec (Mikolov et al., 2013) or SentenceBERT (Reimers and Gurevych, 2019). Some LLMs, such as Llama2 (Touvron et al., 2023) and Mistral (Jiang et al., 2023), have also been made available to the community, whereas others can only be used through a website (sometimes paid) or via an API such as ChatGPT (with models such as GPT-3.5 and GPT-4). The advantages of these closed models lie in their performance and the fact that users do not need computing resources to use them.

## 3 Scoping review and data extraction by experts associated with the MOOD project

The automated processing pipeline presented in this article is designed to enhance and update scoping reviews previously conducted for prototype diseases by the experts associated with the H2020 MOOD project.

A scoping review of the literature was conducted to gather information for a comprehensive understanding of the human, animal, and environmental drivers of three prototype infectious diseases included in the MOOD project: influenza A, leptospirosis, and chikungunya. This review followed a standardized methodology to ensure its quality and repeatability, adhering to the guidelines provided by Tricco et al. (2018).

The key questions were harmonized, and a list of relevant keywords was organized into a standardized search strategy. This strategy was then applied across the following databases: PubMed, Embase, Web of Science, and Scopus.

The inclusion and exclusion criteria were established on the basis of the study design, language (English or other EU languages), time frame (10 years for epidemiological and pathogen data, 30 years for environmental data), geographical location (Europe), and publication type. Studies were excluded if they lacked data, contained non-original or duplicated data (such as reviews, editorials, letters, and modeling studies without data), lacked denominators or reference populations, had unavailable full texts, referred to data from before 2000, or were conducted outside Europe.

The final time frame covered the period from 2000 to 2022. The results of the literature searches for each prototype pathogen were uploaded onto Rayyan (Ouzzani et al., 2016) to select and label the articles according to their main topics of interest (human, environmental, animal, vector, and reservoir covariates). Screening and labeling were conducted independently by three reviewers over two steps: first, by reading titles and abstracts, and then, for the retained articles, by reviewing their full texts. The full texts of the relevant articles were retrieved, and data were extracted via a data extraction sheet based on the template of the Cochrane Consumers and Communication Review Group (Ryan et al., 2018). The extracted data were analyzed to provide basic statistics (numbers and frequencies) for each identified covariate. Table 1 summarizes the study selection steps and the main environmental covariates extracted for all the diseases. The related extraction datasets are available on the Zenodo platform (https://doi.org/10.5281/zenodo.10889957).

**Table 1:** Study selection process and the extracted environmental covariates.

**Disease**	**Number of articles**			**Main environmental covariates extracted**	^**^Frequency
	**Retrieved**	**Included**	^*^ **Selected**		
Influenza A	5,806	453	25	#Humidity	33
				##Temperature	35
Leptospirosis	1,045	95	27	£Time	168
Chikungunya	1,165	65	5	Temperature	24

^*^Extraction of environmental covariate data.

^**^Number of times that the given covariate was extracted from the selected articles.

# Humidity has an impact on virus persistence.

## Temperature has an impact on disease diffusion/spread.

£Time refers to seasonal incidence, i.e., the monthly and weekly distributions of human and animal leptospirosis cases.

## 4 Automation of the scoping review process: method descriptions

In this section, we present a comprehensive overview of the three methods under comparison. These methods align with the three stages delineated in the Preferred Reporting Items for Systematic Reviews and Meta-Analyses (PRISMA) guidelines while considering our aim of updating previously conducted scoping reviews. Therefore, while the planning and preparation phases are not addressed, the three methods focus on article collection and filtering, as well as data extraction. These steps correspond to the search and study selection phases, as well as the data extraction steps outlined in the PRISMA guidelines.

The evaluation and comparison involving the three methods focus on their ability to detect and extract the risk factors (also known as covariates) associated with disease propagation from scientific articles. This task can be conceptualized as an NER task in NLP. Consequently, each word in a given text segment, whether it is a sentence or a small paragraph, undergoes an analysis and is classified into one of two labels: covariate or noncovariate. Notably, to our knowledge, no pretrained language model is capable of accurately identifying words or phrases as environmental risk factors concerning human and animal diseases. Therefore, it is imperative to devise methods for retraining models to effectively perform this task.

1. The first method, which serves as our ***baseline***, entails fine-tuning BERT models to identify covariates within paragraph segments. For model retraining purposes, we utilize a dataset previously annotated by the H2020 MOOD project and presented in the Section 3.
2. Given the limitations of this annotated dataset in terms of comprehensive training, our second method involves performing artificial augmentation via ChatGPT. This augmentation step generates additional sentences with corresponding annotations, thus enriching the dataset. Subsequently, the BERT models are trained on both the original and augmented data, forming our ***hybrid*** approach.
3. Finally, the third method relies solely on ***generative*** models. By leveraging a blend of RAG and few-shot learning, this method provides annotation instances akin to the considered sentence. These annotations guide the model in identifying this new NER category.

Prior to detailing the implementations of these methods, we focus on the preprocessing steps applied to the annotated dataset used for training the models.

### 4.1 Dataset preprocessing for model training

As presented in Section 3, we choose to conduct annotation at two levels, the sentence level, which is very detailed but has a small dataset size, and the entire scientific article level, which does not specify the locations of the identified risk factors in the text. This choice was made because annotation is a time-consuming process, it took about 18 months for all the three diseases and this time frame includes literature screening and consequent data extraction. Furthermore, the task studied here is particularly complex and requires expert annotators in human and animal health, epidemiology, environmental science, and microbiology in line with One Health approach to the diseases. What is more, from a text mining perspective, this task is particularly challenging, as a large portion of the risk factors are found in tables or result figures. In this study, we focused methodologically only on risk factors that were annotated in the body text, which further reduced the size of the dataset used to train the methods. To apply the models to whole articles, we split the text into chunks. We choose to set the size of the chunks to *256* characters to obtain sufficient context for the obtained prediction while guaranteeing a reduced inference time.

The dimensions of the datasets are presented in Table 2. To validate the generalizability of our method, we train the models only on articles concerning influenza. The sentence-level annotations comprise only *20* text excerpts, which we divide into *12* excerpts for training and *8* excerpts for validation. This split of 60% for training and 40% for validation seemed necessary to us given that the dataset is very small.

**Table 2:** Number of scientific articles and 256-character chunks considered.

**Disease**	**Influenza**	**Chikungunya**	**Leptospirosis**	**Total count**
Number of articles	43	7	22	72
Number of chunks	1,372	218	542	2,132
Number of words	52,365	7,885	18,445	78,695

### 4.2 Methodological overviews

#### 4.2.1 Baseline: BERT-like fine-tuning

This approach aims to fine-tune SLM (with 100 of millions of parameters) via the *12* sentences derived from the training dataset. We compare three pretrained models: RoBERTa (Liu et al., 2019) and XLM-RoBERTa (Conneau et al., 2020) (multilingual model) from Meta, as well as BiomedBERT (Gu et al., 2022) from Microsoft. The objective is to evaluate the contribution of multilingualism to this task as well as the specialization exhibited in the biomedical domain relative to RoBERTa, which is a general-purpose language model.

#### 4.2.2 Hybrid method: BERT-like fine-tuning with data augmentation

This second method aims to compare the three previous models, but this time they are trained on an augmented training dataset. Indeed, this dataset has the same *12* original sentences, to which we add *180* chunks artificially created by data augmentation (Nie et al., 2020). This is accomplished via a generative language model: GPT-3.5 from OpenAI (Dai et al., 2023). To achieve this goal, we provide two annotated sentences from the original training dataset in a prompt and ask the model to generate 20 annotations 10 times to obtain a training set of 191 sentences after removing duplicate sentences. The evaluation is performed on the same dataset (*8* chunks).

#### 4.2.3 Fully generative method: RAG with few-shot learning

For this last approach, no pretrained models are fine-tuned. We compare two generative models: GPT-3.5 and GPT-4 from OpenAI. To guide them in this task, we rely on RAG (Lewis et al., 2021). To do this, we compute the embeddings of the 12 sentences obtained from the training set and then store them in an embedding database (FAISS; Douze et al., 2024).

Next, we compare the non-annotated sentence with the training sentences through their semantic proximity (via a calculation of the cosine similarity between their embedding vectors). The 5 annotated sentences that are closest to the sentence to be annotated are injected into a prompt as examples; this process is also known as few-shot learning (Brown et al., 2020). The number of 5 examples is often used in the literature. It balances example diversity well while keeping the prompt context concise. The goal is to provide the models with semantically and grammatically similar sentences that enable them to identify new risk factors contained within the sentences.

### 4.3 Evaluation

The evaluation is conducted at two levels: the sentence and article levels.

#### 4.3.1 Sentence-level annotation

To create this dataset, some sentences from the influenza corpus were annotated to indicate whether they contained covariates, specifying the exact names that appeared in the sentences. No normalization was performed on these annotations. Sentence-level annotation, which is time-consuming, was performed on only 20 sentences from the influenza corpus. Therefore, as presented in this section, we evaluate the trained models on a limited evaluation set (8 sentences) and exclusively for influenza disease.

The metric used is the F1-score as defined below by Equation 1. As shown in Equation 2, Precision, or positive predictive value, is calculated as the ratio of true positives (TP) to the sum of true positives (TP) and false positives (FP). Similarly, Equation 3 defines Recall, or sensitivity, as the ratio of true positives (TP) to the sum of true positives (TP) and false negatives (FN).


(1) $$\begin{array}{c} F_{1}=2\cdot \frac{\text{Precision}\cdot \text{Recall}}{\text{Precision}+\text{Recall}} \end{array}$$

(2) $$\begin{array}{c} \text{Positive Predictive Value}=\text{Precision}=\frac{TP}{TP+FP} \end{array}$$

(3) $$\begin{array}{c} \text{Sensitivity}=\text{Recall}=\frac{TP}{TP+FN} \end{array}$$

For this evaluation, the true or false positive or negative as defined as below:

- TP: The model identifies a correct risk factor.
- FP: The model labels a word but the manual annotation did not select it.
- FN: The model misses a risk factor.

#### 4.3.2 Document-level annotation

The dataset used for this evaluation is described in Section 3. For each scientific article contained in the corpus, the experts provided a list of normalized covariates. For example, if an article states that the rainy periods in April, May, and June are conducive to the persistence of the pathogen, the annotators added the covariate “seasonal precipitation.” This implies that the document-level annotations do not exactly match the expressions that are present in the documents.

The models trained on a subset of the influenza dataset are then applied and evaluated on the full influenza, leptospirosis, and chikungunya corpora. To do this, we divide the entire set of articles into 256-character chunks. Each chunk is then processed by the trained models. Finally, at the end of this inference phase, we compile all the covariates labeled by the models for each document.

To assess the performance of the tested models, we focus solely on the best pair of annotated and predicted covariates for each document. Thus, our evaluation is not exhaustive, as it does not account for false positives or the total number of covariates per document. We focus only on the ability of the models to identify at least one covariate. Since the annotations are normalized, we measure the semantic similarity between the normalized annotations and the words extracted from the text by the models. To do this, we compute the embeddings of these words via the *all-MiniLM-L6-v2* model (Reimers and Gurevych, 2019) and then calculate their similarity levels via the cosine similarity metric. This measure involves calculating the vector product between two embeddings. Its result ranges from 0 (when the compared words have distant meanings) to 1 (when the compared words are synonyms). An embedding is the vector representation of a word in the representation space of a language model.

## 5 Results

The experiments aim to assess three distinct strategies for identifying environmental conditions as risk factors. To accomplish this goal, we conduct evaluations at both the sentence and document levels. Given the two types of available annotations (fine-grained annotations at the sentence level and broader annotations at the document level), our approach involves evaluating the performance of the three tested methods across these two levels. The evaluation conducted at the sentence level offers insights into the training efficacy of the models, whereas the assessment implemented at the document level mirrors the real-world scenarios identified by the H2020 MOOD project, providing a comprehensive overview of covariates identified within a scientific article.

### 5.1 Sentence-level evaluation

The results are reported in Table 3. This table compares the three methods. To compare the performance of the models, the F1 score is used. This metric is the harmonic mean of two other measures, precision and recall, which assess the ability of a model to identify all true positives; the F1 score is lower when false positives are present.

**Table 3:** F1-score produced by the three tested approaches.

**Model**	**Baseline**	**Hybrid**	**Generative**
XLM-RoBERTa-base	0.46	0.52	-
BiomedBERT	0.15	0.14	-
RoBERTa-base	0	0.53	-
GPT-3.5	-	-	0.27
GPT-4	-	-	**0.86**

The best score among all methods is shown in bold font, and the best score yielded by each approach is underlined.

The baseline and hybrid methods are trained for 100 epochs. Although this leads to a high risk of overfitting, given the size of the training dataset, training for only 10 epochs would not allow the models to improve their performance on our task. The overfitting bias of each model is evaluated in the next section.

We observe that the generative approach with GPT-4 outperforms all the other methods. However, it comes with a high cost of $0.05 per analyzed sentence. Indeed, the incurred cost is based on the number of words sent plus the number of generated words. However, the prompts we construct with RAG and few-shot learning are lengthy.

We also observe that implementing data augmentation with the hybrid method improves the performance of the models. Furthermore, RoBERTa-base, which is unable to detect covariates without augmentation, becomes the second-best method through data augmentation (behind GPT-4).

Finally, the poor results of GPT-3.5 stem from the fact that this model hallucinates many covariates from few-shot learning. Moreover, we notice very high variability in its predictions, which makes this model unreliable.

### 5.2 Document-level evaluation

The extraction of risk factors from individual documents is a real-world scenario. Within the entirety of a scientific article, the objective is to identify the environmental factors impacting the dynamics of an epidemic.

To achieve this aim, we perform a series of preprocessing steps. The first step involves extracting textual data from PDF files via the GROBID method.^1^ The advantage of this method is that it also extracts the structure of each PDF document (including its hierarchy of parts and subparts). This aspect is important for filtering only the relevant sections, namely, the abstract, results, and discussion.

We divide the extracted text into 256-character chunks and then apply trained models (with and without data augmentation) and OpenAI models with RAG, i.e., the baseline, hybrid, and fully generative approaches.

This processing pipeline is applied to several scientific articles that have already been annotated by experts. They concern three diseases: influenza, which was used to train the models to conduct detection within the sentences, as well as chikungunya and leptospirosis. Thus, we evaluate the generality of the training process for other diseases in animal healthcare. Table 2 presents the number of scientific articles considered.

To evaluate the ability of the methods to identify covariates in scientific documents, we measure the semantic distances (via the cosine similarity metric) between the embeddings of the manually annotated and automatically extracted covariates. If two words are synonyms, the cosine similarity of their embeddings is close to 1, whereas if two words have completely different meanings, their cosine similarity is close to 0.

We propose to examine two aspects, from the least to the most challenging. The first is to assess whether the methods were able to identify at least one risk factor in the document, as some scientific articles list up to ten. The second evaluation focuses on the methods ability to extract all the risk factors mentioned.

### 5.3 Identification of at least one risk factor

**Figure 1:**
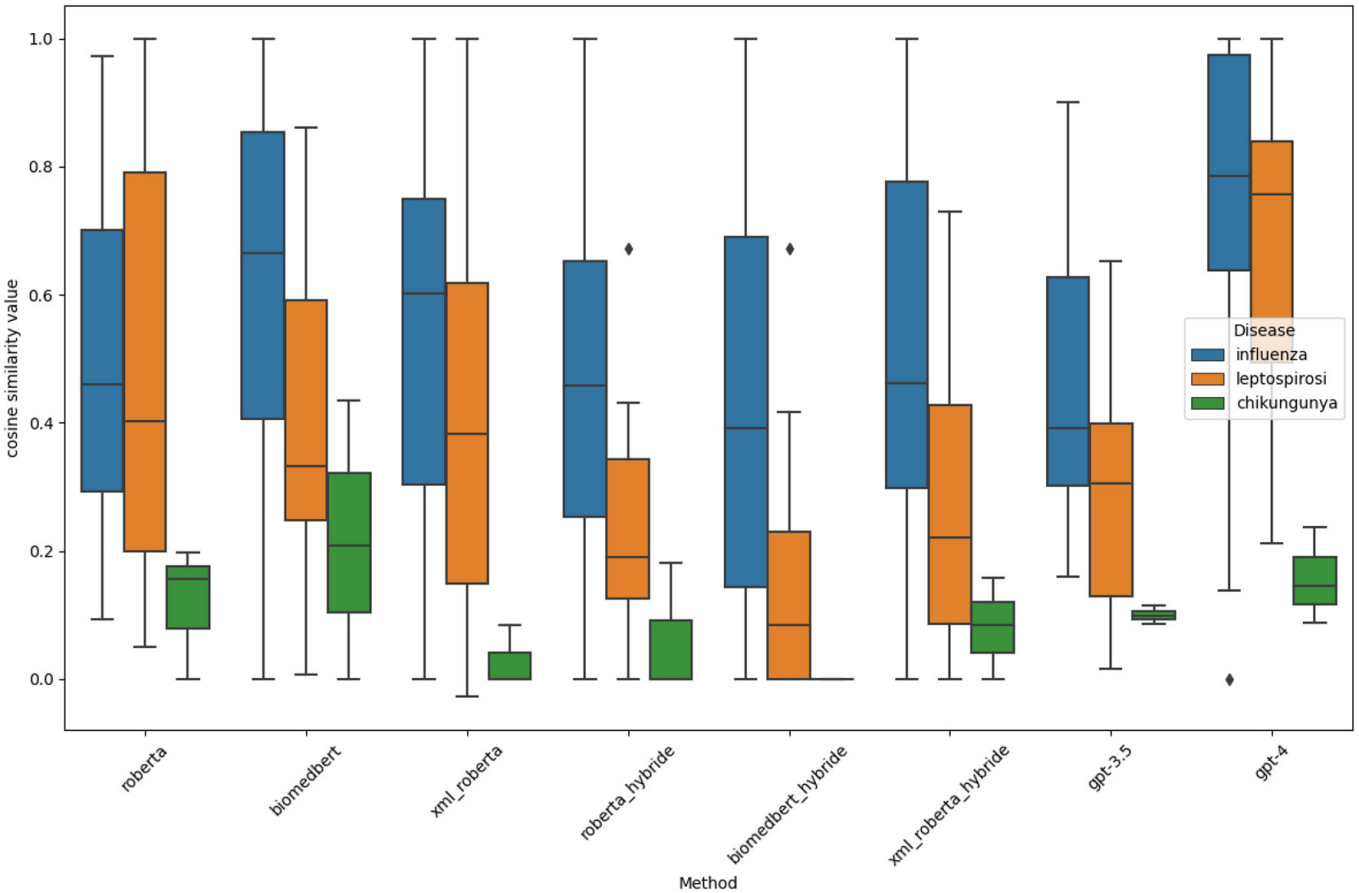
Distribution of the similarity scores between the best pairs of annotated and predicted covariates yielded by the models for each document.

Figure 1 shows the statistical distribution of the cosine similarity scores between the best pairs of annotated and predicted covariates produced for each scientific article concerning each disease. The distribution is presented in the form of a box plot. The line inside each rectangle represents the median of the cosine similarity values, indicating that 50% of the scores are above this value and that 50% are below it. The lower and upper edges of the rectangle indicate that 25% of the scores are below the lower edge and above the upper edge, respectively. For this evaluation, the more compact and closer to 1 a rectangle is, the better the ability of the corresponding method to find at least one covariable. The first observation concerns the spread of all the box plots. This indicates significant variability among the capabilities of the models to identify at least one covariable per scientific paper. The smaller spread for chikungunya is explained by the fact that our dataset contains only 5 articles for this disease. In general, we observe that GPT-4 performs best, with a median close to 0.8, demonstrating its good ability to find at least one covariable. Another observation is that, except for influenza, the hybrid method performs worse than the baseline method. This is likely due to excessive overfitting since the training process is only performed on the influenza data. The final observation is that the models manage to generalize from influenza to leptospirosis, with medians and quartiles that are often close; however, leptospirosis yields worse results, as expected.

### 5.4 Identification of all risk factors

This evaluation aims to study the percentage of risk factors effectively extracted from the entire corpus. The Table 4 shows the results for Leptospirosis, which allows for the evaluation of generalization capabilities to other diseases (similar to the training set). It is clear that only the generative method GPT-4 achieves encouraging scores (10% of risk factors detected). However, this method, when applied to thousands of scientific articles with expert analysis, has the potential to extract new risk factors.

**Table 4:** Evaluation results of leptospirosis.

**Model**	**TP**	**FP**	**FN**	**Precision**	**Recall**	**F1-score**	**% Words found**
RoBERTa_base_*baseline*	2.0	228.0	143.0	0.0087	0.0138	0.0107	1.38
RoBERTa_base_*hybrid*	0.0	114.0	145.0	0.0000	0.0000	0.0000	0.0
BiomedBERT_*baseline*	2.0	222.0	143.0	0.0089	0.0138	0.0108	1.38
BiomedBERT_*hybrid*	0.0	34.0	145.0	0.0000	0.0000	0.0	0.0
XLM_RoBERTA_base_*baseline*	3.0	115.0	142.0	0.0254	0.0207	0.0228	2.07
XLM_RoBERTA_base_*hybrid*	2.0	65.0	143.0	0.0299	0.0138	0.0189	1.38
GPT-3.5	0.0	155.0	145.0	0.0000	0.0000	0.0000	0.0
GPT-4	16.0	722.0	130.0	0.0217	0.1096	0.0362	10.96

**Figure 2:**
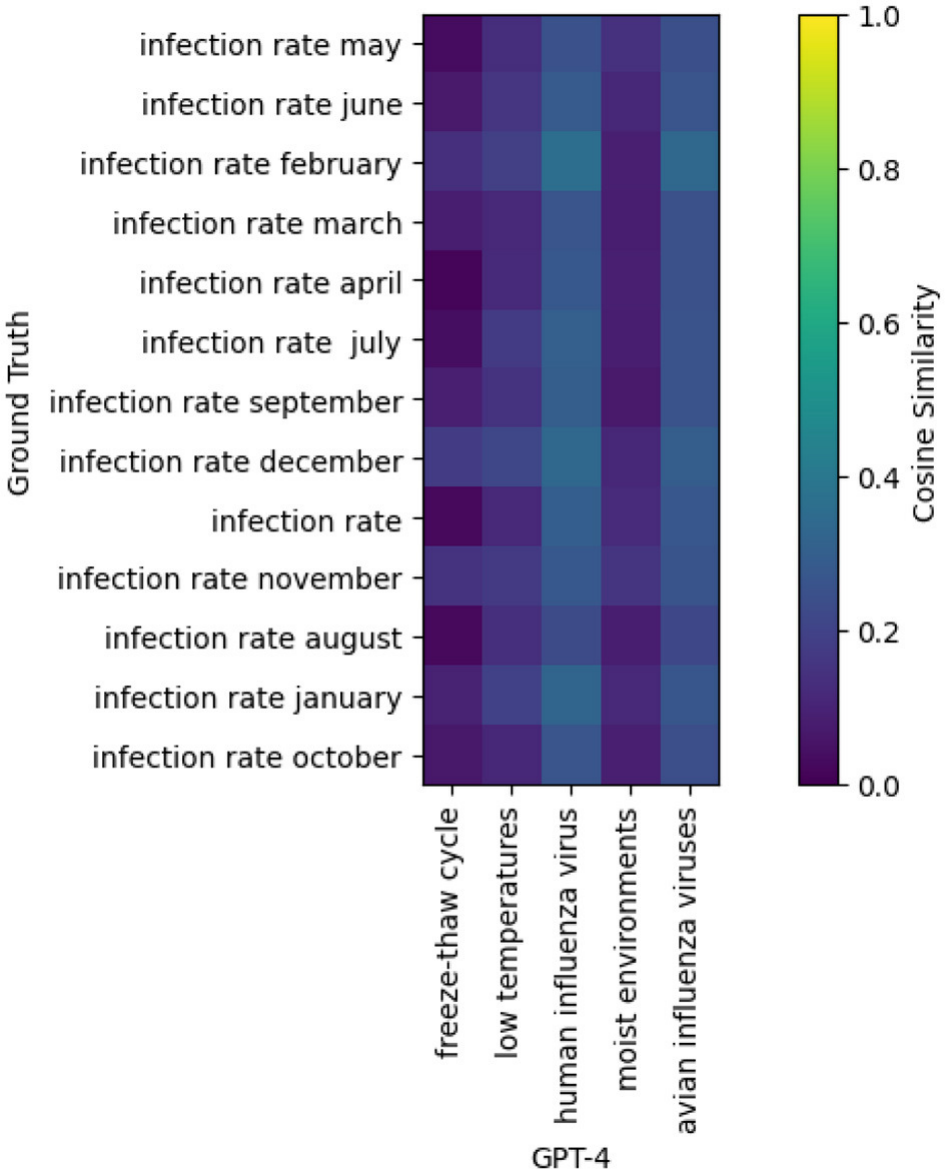
Cosine similarity matrix between the risk factors annotated on the y-axis (“Ground truth”) and the risk factors extracted by GPT-4 on the x-axis, from one of the articles dealing with leptospirosis.

Nevertheless, before using this method operationally, work must be done to address the numerous false positives, which could waste too much time for epidemiologists as shown by Figure 2. In this study, the results focused on sensitivity (i.e., recall), and future work should be conducted on the positive predictive value (i.e., precision).

### 5.5 Method cost

Another factor to consider in the analysis of the results is the cost of using the services of OpenAI. To implement GPT-4 and GPT-3.5 on 72 PDF documents, the costs are $40 and $1. To process the 8,000 articles downloaded for the scoping review, the estimated cost of using GPT-4 is $4,500.

## 6 Discussion

From the results, we have identified four main points. The first point addresses the challenges associated with environmental driver extraction as well as the difficulties encountered when evaluating the results. The second point involves comparing the differences observed between the diseases, highlighting how their specific characteristics impact the results. The third point provides an analysis of the comparisons among the three considered approaches. Finally, we conclude with suggestions for future contributions based on this work.

### 6.1 A challenging evaluation

Automating the environmental driver extraction process is not a trivial task. Depending on the scientific articles considered, the linguistic contexts in which the drivers are mentioned can vary significantly. Some articles compare different drivers and analyse their contributions to the persistence or spread of a pathogen. In such cases, certain drivers may be initially listed but are ultimately dismissed on the basis of the results of the studies. Other articles focus on a single driver and observe the influences of its value ranges in a laboratory setting, whereas some studies examine the spread of a pathogen in the environment. Additionally, to improve readability, some articles do not consistently use the same terminology for drivers throughout the manuscripts (e.g., by using both “precipitation” and “rain”).

These challenges also impact evaluations of the performance of automatic methods. The extracted drivers may not have the exact same names as those annotated by experts. To address this issue, we compare the extracted and annotated drivers via the cosine similarity between their embeddings. Moreover, automatic methods require the input text to be segmented into chunks. It is possible for a candidate driver mentioned in the introduction to be rejected in the results section. However, since a language model does not have access to the entire article, it cannot detect these contradictions.

### 6.2 Differences between diseases

As expected the automated extraction process yields better results for leptospirosis than for chikungunya. Recall that the various models were trained on a dataset composed of articles about influenza, so one might expect the models to perform better when applied to datasets dealing with diseases that share similarities with influenza in terms of their transmission mechanisms.

The poor results obtained for chikungunya can be attributed to two main reasons. First, the manual annotation process considered only indigenous cases in Europe. Since these cases are currently rare compared with cases in tropical regions, they are less frequently described in the considered scientific articles, consequently, the data on environmental drivers were less than the other two diseases. Additionally, their impact directly affects the vector life cycle, activity, and vector competence in transmission of the virus. Conversely, the articles on leptospirosis studied the drivers affecting the persistence of bacteria in their environment, which more closely resembles the influenza dataset.

This is also reflected in the contextualization and scientific language used in the selected studies to describe the environmental drivers, and their importance for the different diseases/pathogens. Although the same driver could have been identified as important for influenza, leptospirosis, and chikungunya, the contextual description of the way the given driver impacts the diseases can be common for some diseases and different for others. In our example, the environmental drivers extracted impacted directly on Influenza and leptospirosis, or indirectly on the habitat/life cycle of the vectors in the case of Chickungunya. The similarities between influenza and leptospirosis were described using the same words, which were not the same for chikungunya (see the example on such terms highligthed in bold in https://github.com/tetis-nlp/automated_scoping_review/tree/main/analysing_results). In conclusion, the performance of extraction among the different methods examined in this study is affected by the amount of similarity of the words used in the articles of Influenza training dataset with the words used in the articles of the other two diseases, as a consequence of the similarity between influenza and leptospirosis, models generalization is better for leptospirosis than for Chikungunya, for which the shared language and terms were lower.

### 6.3 Analysis of the comparison among the tested approaches

The baseline and generative approaches give consistent evaluations at both the sentence level and the document level, whereas the hybrid method performs poorly in terms of the document-level evaluations and exhibits poor transferability when applied to other diseases. The hybrid approach has the advantage of leveraging LLMs while minimizing their financial costs and CO2 emissions since the models are used only during the training phase. However, the hybrid method suffers from significant overfitting. Furthermore, while the annotated sentences extracted from scientific articles tend to be complex in structure and contain precise, measured information, the sentences generated by GPT-3.5 are short, very direct, and lack specificity. Below is a comparison between two sentences:

- **Real sentence:** within this distance range, we estimate that the wind-borne route on its own could explain up to 24% of the new cases.
- **Sentence generated by GPT-3.5:** climate change has been identified as a major risk factor for the spread of infectious diseases.

To mitigate this bias, a larger training dataset is needed to generate more diverse data via large models, along with different epidemiological and linguistic contexts.

**Figure 3:**
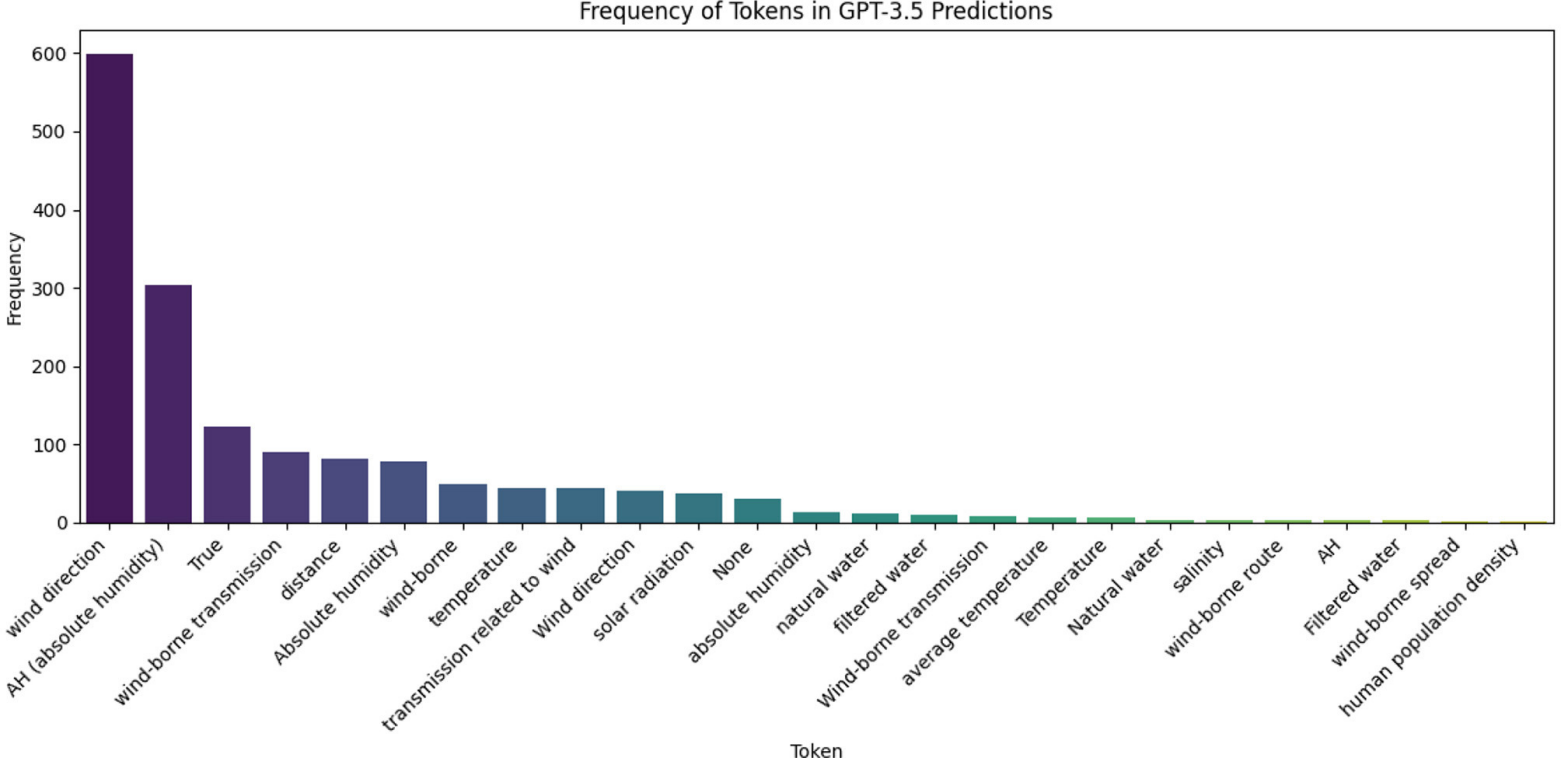
Frequency distribution of risk factors extracted by GPT-3.5 on leptospirosis.

The generative approach produces mixed results depending on the models used. The quality of its extractions depends on two internal factors: the ability of the models to follow our extraction instructions and their knowledge in the epidemiological domain. Indeed, the performance gap between GPT-3.5 and GPT-4 stems from GPT-3.5s struggle to follow the prompt instructions, as it extracts risk factors based on the examples included in the prompt by the RAG. As illustrated in the Figure 3, GPT-3.5 tends to hallucinate, extracting terms like “wind direction” or “absolute humidity,” unlike GPT-4.

As illustrated in the results, GPT-4 achieves encouraging scores, but its cost is a usage barrier in operational contexts or for large-scale literature reviews.

### 6.4 Future works

Given the various points discussed above, two future directions can be considered with respect to the work presented in this article.

The first direction involves improving the baseline and hybrid approaches, which are based on smaller models. To obtain better results, the training datasets need to be expanded. Owing to the poor performance of the hybrid method, artificially generating annotated data remains challenging. However, acquiring these annotations manually is very costly, as doing so requires the involvement of multiple experts. To reduce the time needed for annotation, the methods described in this article can be used on an ongoing basis. They can help identify articles that are likely to contain previously unrecognized drives. After these articles are reviewed and validated by experts, they can be added to the training datasets, thereby reducing the time spent identifying new articles.

The other improvement area focuses on generative methods. This strategy involves comparing models provided by other industry players, who may offer models with capabilities similar to those of GPT-4 but at lower costs.

## 7 Conclusion

Updating scoping reviews or extending them to other regions or diseases is a complex task that requires a significant amount of time. The goal of the present work is to leverage new NLP methods introduced by language models such as BERT and GPT-4. This challenge is approached as a NER task with complex labels and a very limited amount of training data.

We provide two major contributions to accomplish this task. The first includes three datasets derived from a scoping review of the environmental drivers of influenza, chikungunya, and leptospirosis. These datasets enhance the second contribution, which involves training or specializing LLMs or AI systems to extend scoping reviews. We thus assess three classic approaches: fine-tuning models that are now considered small (baseline), the artificial generation of training data to improve the fine-tuning process (hybrid), and finally, the use of LLMs with ground truths and RAG (generative). All the datasets and codes are publicly available.

The results underline that GPT-4 (generative) has encouraging outputs. However, in this context with a very limited amount of training data, data augmentation (hybrid) leads to significant overfitting, preventing the resulting models from effectively generalizing to other diseases.

## Data Availability

The datasets produced and code developed for this study can be found below: datasets: https://doi.org/10.5281/zenodo.10889957, codes: https://github.com/tetis-nlp/automated_scoping_review.

## Footnotes

^1^Grobid. (2008–2024). Available online at: https://github.com/kermitt2/grobid
